# The Inflammatory Role of Serum Amyloid A in the Pathogenesis and Progression of Diabetic Nephropathy

**DOI:** 10.3390/jcm14238427

**Published:** 2025-11-27

**Authors:** Antigoni Stavrou, Christina A. Kousparou, Argyrios Tsakalis

**Affiliations:** 1 Faculty of Life Sciences and Medicine, King’s College London, London WC2R 2LS, UK; antigonistavrou88@gmail.com; 2 School of Medicine, European University Cyprus, 6 Diogenes Street, 2404 Nicosia, Cyprus; c.kousparou@euc.ac.cy

**Keywords:** diabetic nephropathy, serum amyloid A, amyloidosis, inflammation

## Abstract

Diabetic nephropathy (DN) remains the leading cause of end-stage renal disease (ESRD) worldwide, primarily affecting individuals with Type 2 Diabetes Mellitus (T2DM). While traditional risk factors—such as hypertension, poor glycemic control, and dyslipidemia—are well known, recent research has illuminated the pivotal role of inflammation in DN pathogenesis. Inflammatory processes involving chemokines, cytokines, immune cell infiltration, and pro-fibrotic signaling pathways (e.g., NFκB, JAK/STAT) contribute significantly to glomerular and tubulointerstitial damage. Key immune players include macrophages and T lymphocytes, particularly CD4^+^ T cells, which correlate with disease severity and progression. Serum Amyloid A (SAA), an acute-phase reactant traditionally associated with Serum Amyloid A Amyloidosis (AA amyloidosis), has emerged as both a biomarker and active mediator of renal inflammation in DN. SAA promotes cytokine release, leukocyte recruitment, and extracellular matrix remodeling, contributing to glomerular and tubular injury. Elevated Saa3 expression in experimental models correlates with DN progression, while activation of the advanced glycation end products and the receptors for advanced glycation end products (AGE–RAGE) axis in podocytes enhances SAA upregulation and inflammatory signaling. Increasing evidence now indicates that SAA functions, not only as a marker of systemic inflammation, but also as a mechanistically significant driver of intrarenal injury, bridging metabolic dysregulation with sustained inflammatory and fibrotic signaling. Emerging therapeutic approaches—including interleukin 6 (IL-6) blockade, inhibition of AGE formation, targeted anti-fibrotic agents, and recently developed SAA-directed RNA or peptide therapeutics—underscore the therapeutic potential of modulating SAA activity in DN. Preclinical evidence further supports the efficacy of monoclonal antibodies, signaling inhibitors, and dietary anti-inflammatory compounds in mitigating renal injury. Collectively, these developments position SAA as a central mediator at the intersection of metabolic, inflammatory, and fibrotic pathways, highlighting its promise as both a diagnostic biomarker and a therapeutic target for early intervention in diabetic kidney disease.

## 1. Introduction

DN is the leading cause of end-stage renal failure worldwide. While both Type 1 Diabetes Mellitus (T1DM) and T2DM include DN as a complication, the majority of cases of DN are seen in patients with T2DM. Risk factors for DN include modifiable ones, such as hypertension, glycemic level management, dyslipidemia and smoking, as discussed in a paper by Scott et al. Risk factors also include non-modifiable ones such as race, age, family history, genetic profile and sex [1,2].

DN also exhibits a notable hereditary component, reflecting the complex interplay of genetic factors influencing metabolic, vascular, and inflammatory pathways. Several susceptibility genes have been identified, including *ACE*, *APOC1*, and *APOE*, which are involved in lipid metabolism and modulation of the renin–angiotensin–aldosterone system (RAAS), thereby affecting glomerular hemodynamics and oxidative stress responses [2]. Genes such as *GREM1*, *VEGF*, and *HSPG2* contribute to extracellular matrix (ECM) remodeling, angiogenesis, and mesangial expansion, processes central to DN progression. Variants in *eNOS* and *EPO* further influence endothelial function and intrarenal oxygen homeostasis, while *UNC13B*, *CARS*, and *CPVL/CHN2* have been implicated in apoptotic regulation and cellular stress signaling. Additionally, polymorphisms in *ADIPOQ*, *PAI-1*, *TGFβ1*, and *PPARγ* have been shown to modulate oxidative stress, profibrotic activity, and insulin sensitivity, thereby amplifying renal susceptibility in diabetes [3]. Collectively, these genetic associations highlight the multifactorial and polygenic nature of DN, emphasizing the convergence of metabolic dysregulation, vascular injury, and fibrotic remodeling in its hereditary pathogenesis.

There are multiple stages of DN, depending predominantly on the glomerular filtration rate (GFR) and hypertension [2]. In the first stage of DN, after the onset of glomerular basement membrane (GBM) thickening, albuminuria is not present, and GFR is normal. In the final stage of DN, after GBM thickening and mesangial proliferation, progressive glomerular damage leads to elevated microalbuminuria, nodular sclerosis, and ultimately end-stage renal disease (ESRD), which occurs with a GFR below 15 mL min^−1^ per 1.73 m^2^ [2].

As the exact onset of T2DM is not clear, the diagnosis of DN is more challenging. The hallmark of DN is proteinuria. DN is diagnosed using the urine albumin–creatinine ratio (UACR) [4]. Persistent microalbuminuria is defined as 30 mg/g or greater on 2 or more occasions, separated by at least 3 months, using early morning urine samples [4]. In this population, the treatment target to slow CKD progression is a ≥30% reduction in UACR [5]. Urinary biomarkers of tubulointerstitial injury are also involved in the diagnosis of DN [6]. This is important because creatinine changes and albuminuria lack specificity for early tubular injury. Furthermore, evidence suggests that proximal tubular damage may occur earlier than glomerular damage, highlighting the need for tubular biomarkers to improve early detection. Studies have demonstrated the role of neutrophil gelatinase-associated lipocalin (NGAL), kidney injury molecule-1 (KIM-1) and periostin in the earlier diagnosis of DN, and while still mostly available in research settings, these biomarkers show growing translational potential [6].

Management for DN includes optimal glycemic control, alongside treatment for comorbidities including hypertension and dyslipidaemia, which however should be initiated before the onset of diabetic complications, and has reduced efficacy if initiated once complications have occurred. In patients presenting with hypertension, angiotensin-converting enzyme inhibitors and angiotensin receptor blockers are also recommended, due to their additive therapeutic and renal protective properties [4]. Drug therapies with established renal benefit/protection include mineralocorticoid antagonists, sodium-glucose cotransporter-2 inhibitors, glucagon-like peptide 1 agonists, alongside dietary modifications [5]. If ESRD develops, more aggressive routes may be required such as haemodialysis and transplantation.

SAA is a mechanistically significant biomarker and a potential therapeutic target for early intervention and improved outcomes in diabetic kidney disease. This review aims to comprehensively examine the inflammatory role of SAA in the pathogenesis and progression of DN. Specifically, it explores the molecular mechanisms through which SAA contributes to renal inflammation, fibrosis, and functional decline in DN, as well as its potential as a diagnostic and therapeutic target. The purpose of this review is to integrate current evidence to clarify the contribution of SAA to DN and to identify key knowledge gaps that warrant future investigation.

## 2. Inflammatory Paradigm in Diabetic Nephropathy

**Figure 1:**
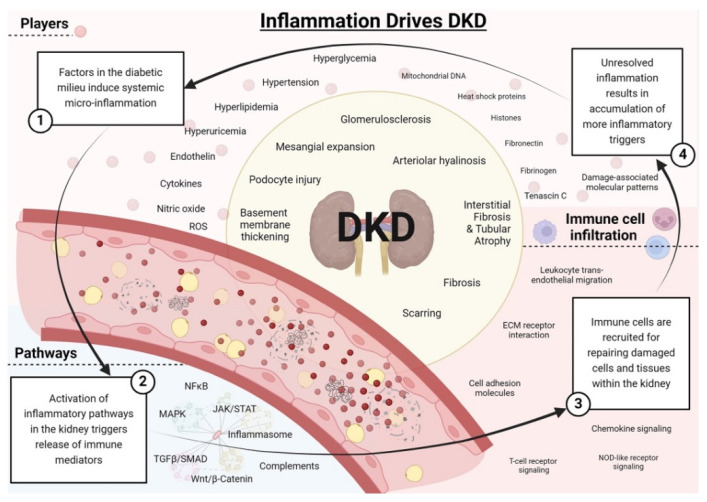
Summary of the role of inflammation in the progression of DN. This figure outlines metabolites and factors in the diabetic milieu and the signaling pathways through which they induce systemic micro-inflammation. It also shows the immune response to kidney damage and how the inflammatory cycle is amplified [8].

Although metabolic and hemodynamic factors substantially contribute to the development and progression of DN, recent studies have suggested that DN is fundamentally driven by chronic, low-grade inflammation [7]. Research has elucidated the role of inflammation in the development and progression of DN, a process involving chemokine production, infiltration of inflammatory cells to the kidney and pro-inflammatory cytokine production. The characteristic histopathological changes observed in DN are glomerular hypertrophy, thickening of the glomerular basement membrane (GBM), and accumulation of extracellular matrix within glomerular and tubular compartments. These changes result in tubulointerstitial and glomerular fibrosis and sclerosis [7]. Evidence has shown that hyalinosis and sclerosis of the larger arteries are relatively common findings in biopsies of patients with T1DM. This is of great importance as clinical observation has shown that around 30–40% of patients with DN do not have significant proteinuria, yet still demonstrate a high prevalence of vascular disease and atherosclerosis, often because of inflammation [8]. Figure 1 summarizes the inflammatory players and pathways which drive DN [8].

Multiple factors involved in diabetes mellitus contribute to the induction of micro-inflammation. These factors include immune cells, T-lymphocytes, cytokines and adhesion molecules. The immune system is a key contributor to DN and includes two branches: the innate and adaptive immune systems [7]. While the adaptive response involves the specific response to a pathogen and the generation of immunological memory, the innate immune response involves the body’s first line of defense against pathogens, providing a non-specific response.

The innate immune response involves macrophages, neutrophils, eosinophils and other early-responding leukocytes. Macrophages are recognized as the principal inflammatory cells involved in the development and progression of kidney damage [7]. There are two subtypes of macrophages: M1 macrophages and M2 macrophages. M1 macrophages are activated by T-helper 1 (Th1) cells and increase the inflammatory response by upregulating pro-inflammatory cytokines, interferon-γ, and reactive oxygen species (ROS). M2 macrophages are activated by T-helper 2 cells and have an anti-inflammatory role by anti-inflammatory cytokine expression, such as interleukin 10 (IL-10) and TGF-β1, hence promoting tissue repairment, remodeling and neovascularization. Given the divergent functions of these macrophage subsets, therapeutic strategies aimed at reducing M1 polarization and enhancing M2-mediated repair may help attenuate the inflammatory cascade in DN, and statins have been proposed as one potential immunomodulatory approach [7].

A key component of the adaptive immune system are T lymphocytes. Th1, Th2, Th17, T reg, and cytotoxic T cells are involved in the development and progression of DN [9]. Emerging evidence indicates that bidirectional crosstalk between infiltrating T cells and activated macrophages amplifies renal inflammation through IFN-γ– and TNF-α–driven activation of NF-κB and JAK/STAT signaling pathways, thereby sustaining cytokine production, enhancing leukocyte recruitment, and perpetuating tubular and glomerular injury. Studies such as that of Nishimura et al. have demonstrated the role of T lymphocytes in adipose inflammation and macrophage recruitment. In diabetic animal models, prior to macrophage accumulation, larger numbers of CD8^+^ effector T cells infiltrated obese epididymal adipose tissue [10]. It was also found that these CD8^+^ T cells promote the recruitment and activation of macrophages, highlighting their essential role in adipose tissue inflammation initiation and maintenance. As CD4^+^ and CD8^+^ T cells can accumulate in the kidney, following their activation they can cause injury to the nephron either directly, through cytotoxic effects, or indirectly, by recruiting and activating macrophages [9]. A study by Han et al. investigated the relationship between CD4^+^ T cells and the development of DN [11]. In this study, 112 adult patients with DN were divided into 2 groups: high-CD4 group (56 patients) and low-CD4 group (56 patients). The results showed that the patients in the high-CD4 group presented with higher proteinuria and lower estimated glomerular filtration rate (eGFR) level than those in the low-CD4 group. Furthermore, renal biopsy showed that patients in the high-CD4 group presented with more severe glomerular lesions, higher density of interstitial inflammation, and more severe tubular atrophy/interstitial fibrosis. These findings underscore the clinical significance of CD4^+^ T-cell infiltration as a contributor to renal inflammation and functional decline in DN [11].

Chemokines are small proteins secreted from immune cells that signal through cell surface G-protein-coupled receptors [12]. Chemokines orchestrate inflammation by recruiting inflammatory cells to the kidney and participate in every phase of kidney injury. One chemokine implicated in the pathogenesis of DN and produced by tubular epithelial cells is monocyte chemoattractant protein-1 (MCP-1). This protein promotes the transformation of monocytes into macrophages. A study by Takebayashi et al. demonstrated that urinary albumin excretion is positively correlated with circulating levels of MCP-1, providing further evidence for its significance in DN pathogenesis [13].

Cytokines are small, biologically active, low-molecular-weight polypeptides secreted by immune cells that influence intercellular communication [14,15]. Inflammatory cytokines exert renal effects related to the expression of adhesion molecules, intraglomerular hemodynamic abnormalities, alterations of the extracellular matrix and basement membranes, apoptosis and necrosis, endothelial permeability, and oxidative stress [15]. An overview of key cytokines involved in renal inflammation is presented in Table 1.

Furthermore, SAA actively participates in renal injury through direct modulation of glomerular, tubular, and endothelial signaling pathways. Experimental studies demonstrate that SAA binds to pattern recognition receptors such as Toll-like receptor 2 (TLR2), Toll-like receptor 4 (TLR4), and formyl peptide receptor-like 1 (FPRL1) on renal cells, triggering downstream activation of NF-κB and Mitogen-Activated Protein Kinase (MAPK) cascades [16,17]. This signaling promotes the transcription of pro-inflammatory cytokines, chemokines, and adhesion molecules, sustaining intrarenal inflammation and leukocyte recruitment [3]. In the diabetic milieu—characterized by hyperglycemia, oxidative stress, and accumulation of advanced glycation end-products—SAA amplifies these pathogenic stimuli by enhancing reactive oxygen species generation and promoting endothelial-to-mesenchymal transition [4]. Moreover, SAA-mediated macrophage activation fosters paracrine injury to podocytes, leading to cytoskeletal disruption, detachment, and eventual glomerulosclerosis—key early events in DN progression [5]. This mechanistic integration highlights SAA as both a driver and amplifier of renal inflammation, linking systemic metabolic dysregulation to local structural and functional injury within the diabetic kidney.

Important inflammatory pathways include the nuclear factor kappa B (NFκB), the Janus kinase/signal transducer and activator of transcription (JAK/STAT) signaling, the MAPK pathway and the complement cascade [8].

The NFκB signaling system is defined by the interactions between NFκB dimers, IκB regulators, and IKK complexes. The NFκB family of transcription factors is a key regulator of inflammation [18]. All resident kidney cells can activate NFκB. It is rapidly activated in diabetic milieu as it is mediated by stimuli including hyperglycemia, AGEs, mechanical stress, ROS, inflammatory cytokines, and angiotensin II. Activation of NFκB occurs when Pattern Recognition Receptors (PRRs) sense pathogen-associated molecular patterns (PAMPS) or damage-associated molecular pattern (DAMPs) and activate a signaling cascade that culminates the freeing of NFκB from its inhibitor IκB and translocate to its nucleus and activate target gene expression. Evidence has shown that in mammal models of DN, the TLR-NFκB pathway is chronically active and promotes glomerular injury. When treated chronically with an NFκB inhibitor, inflammation and expression of the disease decreased [8,19].

The JAK/STAT pathway is a key signaling pathway through which DN is driven. The presence of inhibitory regulators, including the constitutive protein inhibitors of activated STAT (PIAS) and protein tyrosine phosphatases (e.g., PTP1B) and the inducible suppressors of cytokine signaling (SOCS), allow for the transient activation of this pathway [8].

The MAPK pathway and the complement cascade play pivotal roles in SAA-mediated renal inflammation. SAA engages pattern recognition receptors such as TLR2, TLR4, and FPRL1, leading to the activation of MAPK signaling components including ERK1/2, JNK, and p38 MAPK. These kinases promote the transcription of pro-inflammatory cytokines (IL-1β, TNF-α, MCP-1) and adhesion molecules, amplifying glomerular and tubular injury. Concurrently, SAA has been shown to activate the complement system, particularly the C3 and C5 components, thereby enhancing local chemotactic and opsonizing activity within the inflamed kidney. Complement activation products, such as C5a, further stimulate macrophage and mesangial cell responses through cross-talk with MAPK and NFκB signaling, establishing a self-perpetuating inflammatory loop. Collectively, these pathways integrate SAA-driven innate immune signaling with downstream inflammatory and fibrotic processes that characterize diabetic nephropathy [8].

## 3. Serum Amyloid A

Amyloidosis is a rare disorder in which abnormal plasma protein is deposited in tissues, eventually leading to organ dysfunction and death. The most commonly formed proteins include light-chain (in AL), transthyretin (in ATTR) and serum amyloid A (in AA). These proteins tend to form β-pleated sheets, whose antiparallel alignment enables them to form proteolysis-resistant fibrils, ultimately causing cellular and tissue disruption in affected organs [20].

AL is caused by excess light chain production and affects multiple organs, including heart, kidneys, liver, GI tract, peripheral and autonomic nervous system. Associated conditions with AL include multiple myeloma, non-Hodgkin lymphoma and Waldenstrom’s macroglobulinemia. A study has shown that 10–15% of patients with symptomatic myeloma, or with less than 10% bone marrow plasma cells, will also have AL [20].

ATTR amyloidosis is the most common type of hereditary amyloidosis [20]. It is caused by the misfolding of the homotetrameric protein TTR, a protein responsible for transporting thyroxine and retinol-binding protein–retinol complexes in plasma and cerebrospinal fluid [21].

The most common systemic amyloidosis worldwide is AA. SAA exists as multiple different isoforms; including isoforms SAA 1, 2, 3, whose genes are located on chromosome 11p15.1 [22]. Mice express the third SAA isoform, SAA3, which is up-regulated extrahepatically in inflammatory responses and is present in the circulation bound to high density lipoprotein (HDL) [23,24]. Due to the presence of an early stop codon, Saa3 is generally thought to be a pseudogene in humans [25]. Multiple conditions have been associated with AA amyloidosis. It can occur during infectious and non-infectious chronic inflammatory diseases, hereditary periodic fevers, and neoplasms such as Hodgkin lymphoma and renal cell carcinoma, which is the most frequent solid tumour associated with AA amyloidosis. SAA is an acute phase reactant. Cytokines such as IL-1, IL-6 and tumor necrosis factor (TNF) regulate the transcription of SSA proteins, synthesized mainly in hepatocytes. An inflammatory inducer may result in IL-6 increasing the transcription of the mRNA for SAA up to 1000-fold [4]. The SAA that is circulating the body is complexed with HDL. As a result of an inflammatory cascade occurring in the body, apolipoprotein A1 in HDL is displaced by apolipoprotein SAA (apoSAA), which in turn facilitates HDL cholesterol uptake by macrophages [25].

SAA1 and SAA2 are predominantly synthesized by hepatocytes in response to pro-inflammatory cytokines such as IL-1β, IL-6, and TNF-α, representing the major acute-phase reactants in systemic inflammation, but also by macrophages and endothelial and smooth muscle cells. When a high concentration of SAA1 persists for a prolonged period, SAA aggregates into amyloid fibrils, that undergo a process of cleavage, misfolding and aggregation. These isoforms circulate bound to high-density lipoprotein (HDL) and modulate lipid transport, cholesterol efflux, and immune cell chemotaxis. SAA3, in contrast, is primarily expressed in extrahepatic tissues, including adipose tissue, macrophages, renal tubular cells, and intestinal epithelium, suggesting a paracrine or autocrine role in local inflammation.

Human *SAA3* is generally considered a pseudogene, yet its functional analogs are partly recapitulated by *SAA1* and *SAA2* in extrahepatic sites. Within the kidney, SAA1 and SAA3 protein expression has been demonstrated in podocytes, mesangial cells, and tubular epithelial cells, where they promote NF-κB and MAPK activation, cytokine release, and matrix remodeling. In the vasculature, SAA1/2 induce endothelial adhesion molecule expression, facilitating leukocyte recruitment and vascular inflammation. In adipose tissue, SAA3 regulates macrophage infiltration and insulin resistance through TLR2/4-mediated pathways. Collectively, these isoforms form a coordinated network that links systemic and local inflammatory signaling, lipid metabolism, and tissue remodeling across multiple organs [26,27].

Among the SAA isoforms, SAA1 and SAA2 are most prone to misfolding and fibrillogenesis, while SAA3, expressed locally in renal tubular cells, may further amplify parenchymal aggregation, together promoting glomerular and interstitial deposition in the kidney. The kidney is particularly susceptible to SAA amyloid deposition due to its high blood flow and the filtration function of glomeruli, which expose the glomerular basement membrane (GBM) and mesangial matrix to circulating SAA. Negatively charged extracellular matrix components in the GBM and mesangium facilitate binding and nucleation of amyloid fibrils. Chronic inflammatory stimuli, oxidative stress, and advanced glycation end-products promote SAA misfolding, enhancing local aggregation. Consequently, SAA preferentially deposits in glomeruli and tubular interstitium, leading to proteinuria and progressive renal injury.

Acute phase SAA is believed to not only present as an inflammatory biomarker, but also as an active immune effector. These roles include promoting the secretion of inflammatory mediators such as IL-1β, IL-4, IL-8, COX-2 and TNF-α, the cell differentiation of Th17 cells, and hence initiation of adaptive immunity. It has been observed that rheumatoid arthritis patients with elevated levels of local SAA have increased matrix metalloproteinase (MMP), which further damages the joint [26].

SAA is also involved in generating superoxide production and promoting inflammatory cell infiltration and migration, activating innate immune cells via surface receptors and directly activating the inflammasome. Specifically, it exerts pro-inflammatory effects by engaging receptors TLR2, TLR4, FPR2, and RAGE. Activation of TLRs and RAGE triggers NF-κB and MAPK signaling, leading to cytokine and chemokine production, while FPR2 mediates immune cell chemotaxis. This receptor-mediated network links circulating SAA to local tissue injury and fibrosis in the kidney.

**Figure 2:**
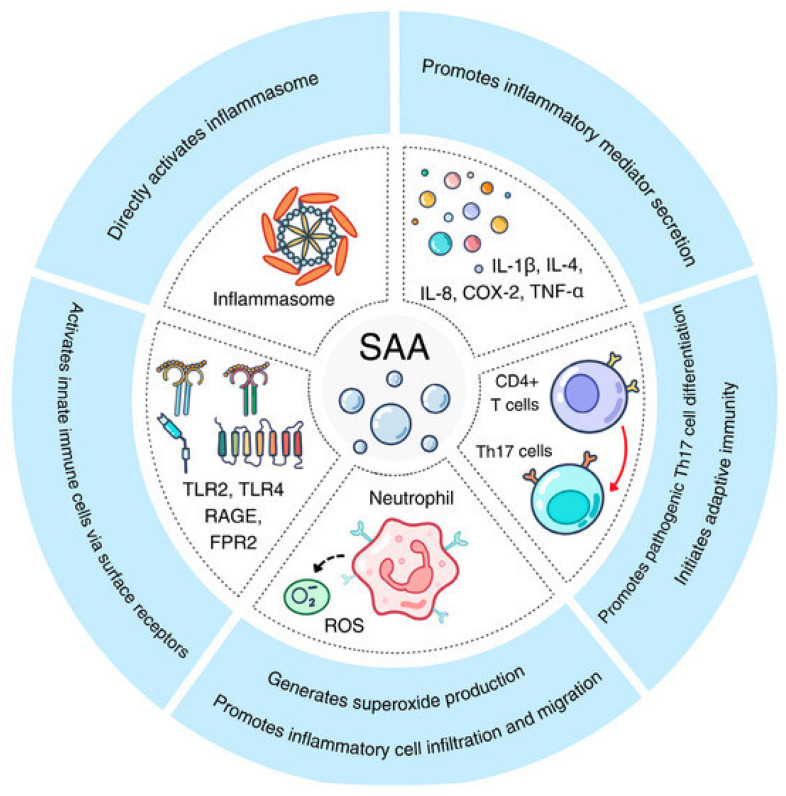
Summary of the proinflammatory pathways activated by acute-phase SAA. These involve generation of superoxide production, activation of innate immunity via cell surface receptors and direct activation of the inflammasome. Inflammatory mediator secretion and Th17 differentiation are also promoted, leading to the initiation of adaptive immunity [28].

Studies have been conducted to demonstrate the role of SAA in inflammation by genetically altering mice to reduce their SAA1, SAA2 or SAA3 levels. Inflammation was reduced in these disease models, which highlights the role of SAA in inflammation [24]. Figure 2 provides a summary of the proinflammatory activities and their underlying mechanisms [28].

## 4. Serum Amyloid A in Diabetic Nephropathy

Early detection of SAA levels in circulation is feasible through high-sensitivity immunoassays, which can identify subclinical elevations preceding overt organ involvement. Early SAA elevation is typically asymptomatic but reflects ongoing cytokine-driven inflammation and correlates with increased risk for progressive renal involvement. Although early rises in SAA do not produce direct clinical symptoms, they serve as a sensitive biomarker of disease activity and an early warning signal for potential amyloidogenesis. Preventive strategies focus on strict control of the underlying inflammatory process through disease-modifying drugs, biologic agents targeting IL-1, IL-6, or TNF-α, and early institution of anti-inflammatory therapy to normalize SAA levels. Regular monitoring of circulating SAA concentrations in high-risk patients enables timely therapeutic adjustment, thereby reducing the likelihood of SAA-driven renal injury and subsequent amyloid deposition.

Early functional renal changes in SAA-associated pathology encompass subtle but clinically significant disturbances in glomerular hemodynamics and tubular function. A modest decline in GFR may represent the first measurable perturbation, indicating early compromise of glomerular autoregulation and endothelial homeostasis. Microalbuminuria frequently follows as a sensitive marker of glomerular barrier dysfunction and podocyte stress prior to the development of overt proteinuria. Progressive accumulation of SAA-derived amyloid fibrils within the mesangial matrix and glomerular basement membrane subsequently exacerbates permeability defects and accelerates GFR decline [5]. Parallel to glomerular involvement, experimental and clinical studies have demonstrated that SAA and its oligomeric intermediates exert direct cytotoxic and pro-inflammatory effects on tubular epithelial cells, leading to oxidative stress, mitochondrial injury, and impaired re-absorptive function. These early tubular derangements manifest as low-molecular-weight proteinuria and mild electrolyte abnormalities, reflecting impaired solute handling at the proximal tubular level. Activation of innate immune pathways, including toll-like receptor and NLRP3 inflammasome signaling, further amplifies interstitial inflammation and fibrogenic responses [8]. Human studies corroborate these mechanistic observations, showing that elevated circulating SAA levels correlate with early reductions in GFR and subtle increases in urinary protein excretion even in the absence of established amyloid deposits. Renal biopsies from patients with chronic inflammatory conditions have revealed early SAA deposition, mesangial expansion, and podocyte effacement preceding mature fibril formation, suggesting that soluble or misfolded SAA species serve as nephrotoxic intermediates [8].

Importantly, these early functional changes may be partially reversible with effective suppression of systemic inflammation and consequent reduction in SAA levels. Collectively, these findings support a biphasic model of SAA-associated renal injury characterized initially by reversible, functionally mediated disturbances in glomerular and tubular physiology, followed by irreversible structural remodeling and amyloid deposition. Early recognition and intervention at this functional stage are critical to preserving nephron integrity and mitigating progression toward overt amyloid nephropathy.

Specifically as to the role of inflammation, it has been identified as a key molecular driver of DN [29]. Hence, studies have been conducted to investigate a link between AL and DN, and the role of SAA as a potential mediator of DN.

A study by Saliu et al. was conducted to investigate the progression of DN in mice models and the use of bioluminescence imaging to visualise pathophysiological changes in the kidney that characterize DN non-invasively. Results show Saa3, which is increased 5 times in one of the renal models, is a significant biomarker, and monitoring its activity could be useful in evaluating the degree of kidney damage and progression of DN [29].

The AGE-RAGE interaction in podocytes increases SAA, suggesting that SAA may promote glomerular inflammation. AGEs are harmful compounds formed by non-enzymatic glycation, a process where proteins or fats combine with sugars (like glucose) in the blood. AGEs accumulate more rapidly in chronic hyperglycaemia, resulting in renal function impairment, as the kidney is the major site of AGE clearance [30]. The compounds can make tissues stiffer by cross-linking proteins, damage the extracellular matrix and bind to cellular receptors and trigger inflammation and oxidative stress. These RAGEs are cell surface receptors found on cells such as podocytes, endothelial cells and immune cells [31,32]. Podocytes are terminally differentiated cells of the kidney glomerulus that are essential for the integrity of the kidney filter. Their function is primarily based on their intricate structure, which includes foot processes. Loss of these actin-driven membrane extensions is tightly connected to the presence of protein in the urine, podocyte loss, development of CKD, and ultimately renal failure [33]. The role of AGEs in the development of DN has been investigated in studies targeting the AGE-RAGE pathway, which have shown that AGE formation inhibitors such as aminoguanidine reduce renal pathological changes [30].

What is important to highlight is that SAA further amplifies NF-κB and MAPK signaling in resident renal cells and infiltrating leukocytes, creating a feed-forward loop that promotes inflammation, extracellular matrix deposition, and progressive glomerular and tubular injury. This AGE–RAGE–NF-κB–SAA axis represents a mechanistically informed target for early intervention and biomarker-guided management in diabetic kidney disease.

Integrin-linked kinase (ILK) is a key mediator of cytoskeletal organization, cell adhesion, and extracellular matrix remodeling in renal cells, processes that are central to the progression of diabetic nephropathy [34]. Current evidence suggests that ILK upregulation can occur both downstream of SAA signaling and independently in response to hyperglycemia, TGF-β, mechanical stress, or AGEs. SAA activates NF-κB and MAPK pathways, which in turn enhance ILK expression in podocytes and tubular epithelial cells, linking systemic inflammation to structural and functional alterations in the kidney. Similarly, a study by Cheng et al. demonstrated that treatment of podocytes with AGEs reduced cell adhesion and increased ILK levels, indicating impaired podocyte function via ILK upregulation and RAS activation [35]. However, ILK is also responsive to non-SAA stimuli, including glucose-induced oxidative stress and integrin-mediated mechanotransduction, suggesting that its upregulation is not exclusively dependent on SAA. Collectively, these findings indicate that ILK functions as a convergent signaling node integrating multiple upstream pathways—both SAA-dependent and -independent—contributing to cytoskeletal remodeling, podocyte dysfunction, and fibrogenesis in diabetic kidney injury. Clarifying the relative contributions of these pathways remains critical for understanding ILK’s potential as a therapeutic target.

## 5. Future Therapeutic Approaches

Beyond elucidating mechanistic dimensions, the novelty of this review lies in its conceptual integration of SAA as a unifying molecular nexus that bridges metabolic, inflammatory, and fibrotic pathways in DN. By examining SAA’s dual role as a dynamic inflammatory effector and potential therapeutic target, the discussion moves beyond descriptive cataloguing to propose a mechanistically cohesive framework for understanding disease progression. Recent preclinical studies have demonstrated that pharmacologic modulation of SAA activity—via IL-6 or TNF-α blockade, TLR inhibition, or direct neutralization of circulating SAA—attenuates renal inflammation, oxidative stress, and proteinuria in diabetic models [36,37]. These findings underscore the translational potential of targeting SAA not merely as a biomarker of disease activity but as an actionable mediator in the pathogenic continuum of DN. Collectively, this section reframes SAA as an active driver of renal injury and highlights the therapeutic rationale for targeting SAA-associated pathways at multiple levels of the inflammatory cascade. The fact that SAA and inflammation play a role in the development of DN, directs us to focus on anti-inflammatory therapies alongside optimal glycemic control for the prevention and management of DN [38]. These include therapies that inhibit the formation of AGEs, or AGE crosslink breakers, such as Pyridoxamine (PDX), a derivative of vitamin B_6_ [39]. Clinical trials have supported that diet modification and anti-inflammatory supplementation such as vitamin D and curcumin could be useful in reducing inflammation [40,41,42].

However, despite these promising findings, current therapeutic strategies remain limited by several key challenges. These include reliance on small and heterogeneous patient cohorts, the broad immunomodulatory nature of many agents that predisposes patients to off-target effects, and the scarcity of renal-specific clinical trials adequately powered to evaluate kidney outcomes. These limitations underscore an urgent need for more selective, kidney-focused, and biomarker-guided interventions.

Newly studied therapies—including monoclonal antibodies, signaling pathway inhibitors, peptide-based amyloid disruptors, and anti-fibrotic agents—are showing preclinical promise and are moving towards clinical trials. Tocilizumab, an antibody blocking the IL-6 receptor, has stabilized or improved the kidney function of patients with renal amyloidosis, more so in patients with active inflammation and CRP above the baseline. Larger randomized studies need to be conducted to establish the efficacy and safety of these therapies [43,44,45,46].

Of particular interest are emerging SAA-targeted RNA therapeutics—such as small interfering RNA (siRNA) and antisense oligonucleotides designed to suppress hepatic SAA synthesis—which offer the potential for highly selective repression of SAA production at its source. Additionally, engineered peptide constructs capable of binding SAA, inhibiting fibrillogenesis, or destabilizing existing oligomers represent an innovative therapeutic class aimed at interrupting early amyloidogenic processes. These platforms, though still in early development, aim to overcome current therapeutic limitations and may ultimately enable kidney-specific, mechanism-directed modulation of SAA biology.

Together, these emerging therapies illustrate an evolving landscape in which SAA-directed strategies may complement existing anti-inflammatory, anti-fibrotic, and metabolic interventions to provide more precise and durable renoprotection in DN.

## 6. Concluding Remarks

Collectively, this analysis advances a novel interpretive perspective by integrating molecular mechanisms, experimental evidence, and therapeutic implications, thereby providing a platform for the development of SAA-centered diagnostic and interventional strategies in diabetic kidney disease. Future research should prioritize mechanistic mapping of SAA signaling networks, development of renal-targeted delivery systems, and large-scale clinical studies powered to evaluate SAA-specific therapeutic benefit. Expanding these therapeutic strategies may ultimately contribute to improved clinical outcomes and help prevent kidney function decline in individuals with SAA-driven renal pathology.

## Figures and Tables

**Table 1:** This table provides an overview of the cytokines linked to inflammation in the kidneys.

Molecule	Source/Activation	Key Actions	Role in DN
IL-1	Produced by immune & kidney cells; increased in DM models	Increases adhesion molecules (ICAM-1, E-selectin), induces prostaglandin E2, hyaluronan synthesis	Promotes inflammation, alters glomerular hemodynamics, increases ECM synthesis, linked to albuminuria
IL-6	Produced by many cell types; increased in DN patients	Modifies ECM dynamics, promotes cell proliferation, increases vascular permeability	Leads to membrane thickening, ECM overproduction, DN progression
IL-18	From T-cells, macrophages, monocytes, tubule cells	Upregulates other cytokines, increases ICAM-1, induces endothelial apoptosis	Correlates with albuminuria and nephropathy severity
TNF-α	Produced by infiltrating immune cells & kidney cells	Activates transcription factors, adhesion molecules; direct cytotoxic effect; alters hemodynamics	Drives inflammation, ROS production, ECM buildup, hypertension; correlates with albuminuria
TGF-β1	Produced by kidney cells	Phosphorylates Smads, promotes ECM production, inhibits ECM degradation, induces fibroblast formation	Major mediator of renal fibrosis & sclerosis; active form drives damage; latent form protective
USF1 & USF2	Transcription factors in the Myc family	Regulate glucose-responsive genes	Overexpression linked to albuminuria & DN progression
Smads	Intracellular proteins activated by TGF-β1	Smad2/3 bind Smad4 → gene transcription; Smad7 inhibits pathway	Smad4 controls inflammation; Smad7 inhibits fibrosis—loss of Smad7 leads to persistent fibrosis

## Data Availability

No new data were created or analyzed in this study. Data sharing is not applicable to this article.

## Abbreviations

The following abbreviations are used in this manuscript:

AA	Amyloid A amyloidosis
AL	Light chain amyloidosis
ATTR	Transthyretin amyloidosis
DN	Diabetic Nephropathy
ESRD	End-stage renal disease
GFR	Glomerular filtration rate
GBM	Glomerular basement membrane
UACR	Urine albumin–creatinine ratio
NGAL	Neutrophil gelatinase-associated lipocalin
KIM-1	Kidney injury molecule-1
GLP-1	Glucagon-like peptide-1
Th1	T-helper 1
ROS	Reactive oxygen species
TGF-β1	Transforming Growth Factor Beta-1
MCP-1	Monocyte chemoattractant protein
IL-1	Interleukin-1
ILK	Integrin-linked kinase
TNF-α	Tumor Necrosis Factor-alpha
USF1	Upstream Stimulatory Factor 1
NFκB	Nuclear factor kappa B
MAPK	Mitogen-Activated Protein Kinase
PRRs	Pattern Recognition Receptors
PAMPS	Pathogen-associated molecular patterns
AGEs	Advanced-glycation end products
RAGE	Receptors for advanced glycation end-products
JAK/STAT	Janus kinase/signal transducer and activator of transcription
HDL	High-density lipoprotein
GAGs	Glycosaminoglycans
MMP	Matrix metalloproteinase
COX-2	Cyclooxygenase-2
PDX	Pyridoxamine
CRP	C-reactive protein
